# Transcript Analysis Reveals a Hypoxic Inflammatory Environment in Human Chronic Otitis Media With Effusion

**DOI:** 10.3389/fgene.2019.01327

**Published:** 2020-02-21

**Authors:** Mahmood F. Bhutta, Jane Lambie, Lindsey Hobson, Debbie Williams, Hayley E. Tyrer, George Nicholson, Steve D.M. Brown, Helen Brown, Chiara Piccinelli, Guillaume Devailly, James Ramsden, Michael T. Cheeseman

**Affiliations:** ^1^ Department of ENT, Brighton & Sussex University Hospitals NHS Trust, Brighton, United Kingdom; ^2^ Nuffield Department of Surgical Sciences, University of Oxford, John Radcliffe Hospital, Oxford, United Kingdom; ^3^ Department of ENT, Oxford University Hospitals NHS Trust, John Radcliffe Hospital, Oxford, United Kingdom; ^4^ Mammalian Genetics Unit, MRC Harwell Institute, Harwell, United Kingdom; ^5^ Faculty of Health and Wellbeing, University of Lancashire, Preston, United Kingdom; ^6^ Department of Statistics, University of Oxford, Oxford, United Kingdom; ^7^ The Roslin Institute, University of Edinburgh, Midlothian, United Kingdom; ^8^ The Royal (Dick) School of Veterinary Studies, University of Edinburgh, Midlothian, United Kingdom; ^9^ GenPhySE, Université de Toulouse, INRA, ENVT, Castanet Tolosan, France

**Keywords:** otitis media, effusion, transcript, inflammation, hypoxia

## Abstract

Chronic otitis media with effusion (COME) is the most common cause of childhood hearing loss in the developed world. Underlying pathophysiology is not well understood, and in particular the factors that lead to the transition from acute to chronic inflammation. Here we present the first genome-wide transcript analysis of white blood cells in the effusion of children with COME. Analysis of microarray data for enriched pathways reveals upregulation of hypoxia pathways, which is confirmed using real-time PCR and determining VEGF protein titres. Other pathways upregulated in both mucoid and serous effusions include Toll-like receptor signaling, complement, and RANK-RANKL. Cytology reveals neutrophils and macrophages predominated in both serous and mucoid effusions, however, serous samples had higher lymphocyte and eosinophil differential counts, while mucoid samples had higher neutrophil differential counts. Transcript analysis indicates serous fluids have CD4+ and CD8+ T-lymphocyte, and NK cell signatures. Overall, our findings suggest that inflammation and hypoxia pathways are important in the pathology of COME, and targets for potential therapeutic intervention, and that mucoid and serous COME may represent different immunological responses.

## Introduction

Otitis media with effusion (OME) or “glue ear” is the most common cause of hearing loss in children in a developed world environment. The disease is characterized by an inflammatory effusion that prevents transmission of sound through the middle ear space, and if chronic (chronic OME, COME) is associated with potential effects on language acquisition and learning.

The pathophysiology underlying OME is not fully understood. It often follows an episode of acute otitis media (AOM), where ascent of bacteria from the nasopharynx leads to a purulent effusion in the middle ear. As this purulent effusion resolves, the serous or mucoid effusion of OME replaces it. The effusion of OME is usually short-lived, but in a minority of cases the effusion persists as COME (defined as OME of duration >3 months). COME affects 5–6% of all children in their second year of life (Bhutta, 2014), and if associated with hearing disability may be treated with the surgical insertion of grommets (ventilation tubes) which eliminate the effusion.

Data from mouse models demonstrate differing expression of cytokines in the middle ear, and differing involvement of leucocytes and epithelial cells, through the onset, development, and resolution of an episode of AOM (Hernandez et al., 2015). Expression of inflammation-associated genes has also been found in peripheral blood mononuclear cells in children with AOM (Liu et al., 2012; Liu et al., 2013). However, the molecular events that may be pivotal in the transition from acute to chronic inflammation in the middle ear are not known (Bhutta et al., 2017). The high heritability of time with middle ear effusion (Casselbrant et al., 1999) suggests that host response is relevant.

This question is important. Unlike some chronic inflammatory disorders, chronic otitis media can and usually does improve in later life (Caye-Thomasen et al., 2008). In addition COME is sometimes a risk factor for more serious ear disorders such as cholesteatoma (Jennings et al., 2017). An understanding of what causes chronic inflammation in the middle ear could therefore allow us not only to understand what causes disease but could also highlight potential molecular targets to affect earlier and more reliable resolution. To date molecular analysis of the effusion in COME has been fragmentary.

Proteomic analysis of effusions from children undergoing grommet surgery shows abundance of innate immunity products of neutrophil leukocytes, neutrophil extracellular traps (NETs), epithelial/glandular anti-microbial proteins (such as BPIFB1, also known as LPLUNC and BPIFA1, also known as SPLUNC) and mucins such as MUC5B (Val et al., 2016). Cytokine analysis reveals a wide spectrum of pro-inflammatory cytokines, among which is IL-8 the neutrophil leukocyte chemotaxic protein (Matkovic et al., 2007; Val et al., 2016). Hypoxia signaling pathway members such as vascular endothelial growth factor (VEGF) protein and mRNA are also present in the effusion and middle ear mucosa (Jung et al., 1999; Sekiyama et al., 2011; Val et al., 2016; Val et al., 2018).

The effusion in younger patients with COME is predominantly mucoid rather than serous (Duah et al., 2016). There are microbiome difference between mucoid and serous effusions (Krueger et al., 2017), and proteomic analysis reveals a higher content of MUC5B in mucoid effusions, as well as DNA (Val et al., 2018). Mucoid effusions also have a global tendency to increased pro-inflammatory mediators, but serous effusions have greater acute phase proteins IL-1b and IL-10, suggestive of a differing immunological response (Val et al., 2018).

In this study we perform the first whole genome transcript analysis of white blood cell (wbc) gene expression from effusions in children with COME. We compare samples with peripheral venous blood as a baseline, and then make comparisons of serous versus mucoid effusions. We also examine the cytology of such effusions.

We report that microarray analysis of effusions showed upregulation of hypoxia signaling, response to hypoxia, Toll-Like Receptor signaling, the complement and the RANK-RANKL pathways. RTqPCR showed upregulation of hypoxia signaling genes and VEGF protein titres were elevated. Mucoid and serous effusions had different cytological profiles that parallel differences in T-lymphocyte, NK cell and myeloid cell signatures in the microarray analysis.

## Materials and Methods

### Ethics Statement

This study was approved by Oxfordshire Research Committee C (reference 11/SC/0057). Informed consent was obtained from the child's guardian for the collection, retention and analysis of samples. All patient records and associated metadata (age, racial ethnicity, duration of disease, vaccination history, antibiotic use, previous grommet surgery, and occurrence of atopy) were anonymized.

### Chronic OME Phenotypes

Children with COME were diagnosed using standard clinical and audiological tests that met the UK National Institute for Health and Care Excellence guidelines for surgical management of glue ear (http://publications.nice.org.uk/ifp60). Clinical samples were collected under general anesthesia from children undergoing grommet (ventilation tube) insertion. Syndromic children were excluded. No children had been given glucocorticoid steroids or antibiotics in the three months prior to surgery.

### Sample Collection

A 3.5 ml venous blood sample was collected from the hand vein using a BD vacutainer and 1ml transferred to a lithium heparin tube (Kabe LaborTechnik GmbH) for plasma sample preparation and 2.5 ml Intracellular RNA from blood was isolated with PAXgene Blood RNA kit (PreAnalytiX, Qiagen) following manufacturer's instructions.

Individual COME fluid samples from each middle ear were collected through a myringotomy using a Juhn Tym-Tap® aspirator/collector device (Medtronic). The surgeon evaluated the gross appearance and viscosity of the effusion at time of surgery and characterized the aspirate as serous, mucoid or intermediate, and noted whether or not the sample was blood-stained.

A cytology preparation was made (see below) and the remainder was snap frozen on dry ice and stored at −70°C.

### Glue Ear Cytology

Forty-eight cytology smears were made from 30 children (one ear sample per child), and 9 children (samples from both ears). Material adherent to the aspirator tube was used to make a cytology smear on a glass slide. These were methanol fixed and Giemsa stained and graded for mucus; red blood cells, and overall cellularity, white blood cell differential performed on 200 cells; leukocyte morphology; presence of bacteria and epithelial cells. The criteria used for grading the samples are given in **Table S1**. The patient ID and effusion phenotype was blinded for evaluation.

Mixed models were used to compare the leukocyte proportions between the mucoid categories (serous, intermediate and mucoid treated as ordinal). Patients were fitted as random effects and mucoid category as a fixed effect. These models take into account any correlation occurring between measurements taken from the ears of the same children. Each type of leucocyte (i.e. lymphocytes, neutrophils, etc.) was analyzed using a separate mixed model. Mixed models with the same structure were used to compare leukocyte measurements between other categories (e.g. bloody ear, first grommet, and demographic characteristics).

Cell cytological characteristics (macrophage vacuolation, macrophage erythrophagocytosis) were analyzed using mixed ordinal regression models. Again patient effects were fitted as random to allow for any correlation between the results from ears of the same children. Generalized linear mixed models (GLMMs) were used to examine associations between binary outcomes (e.g. bloody ear) and other characteristics. In situations where data were very sparse and the model did not converge, then a Fisher's exact test was used. However, this takes no account of the potential correlation between data from the ears from same children.

### RNA Isolation

Intracellular RNA from blood was isolated with PAXgene Blood RNA kit (PreAnalytiX, Qiagen) following manufacturer's instructions and its quality was assessed using a bioanalyzer (Agilent Tecnologies).

RNA from middle ear effusions was extracted using the NucleoSpin® kit (Macherey-Nagel) following manufacturer's instructions. Due to the large volume of some effusions the initial volume of lysis buffer and β-mercaptoethanol was increased to 1 ml and the sample run through the tube through several rounds of centrifugation. Due to the larger sample volumes at this initial stage, flow-through was collected in 15 ml tubes.

### cDNA Synthesis

cDNA was generated following manufacturer's instructions using a high capacity cDNA reverse transcriptase kit containing MultiScribe™ Reverse Transcriptase (Applied Biosystems). Samples were either used immediately in RT-qPCR reactions or stored long term at −80°C.

### Transcriptional Profiling

Transcriptome analysis was performed on 6 serous COME effusions from six children and 6 mucoid effusions from five children (samples from both ears in one child), and their matching 11 blood samples. Transcriptional profiling was performed on an Affymetrix GeneChip® Human Gene 2.0 ST Array platform. The selection of samples was based on the quantity and quality of the RNA sample judged by RNA integrity number (RIN). In the first experiment 6 mucoid samples (RIN 6.1-7.2) and corresponding patient blood samples were analyzed. In the second 6 serous samples (RIN 6.4-7.7) and corresponding blood sample. Two of the serous ear samples came from the right and left ear of the same child. Corresponding blood samples from these patients RIN scores ranged from 8.1–9.1

### Microarray Analysis

CEL files were processed and normalized using AromaAffymetrix (https://statistics.berkeley.edu/tech-reports/745) and a chip definition file provided by Brainarray (Dai et al., 2005). Differentially expressed genes were found using limma (Ritchie et al., 2015) with an adjusted *P*-value cut-off of 0.01 and a minimal difference of a factor 2 (ie absolute value of the log2 of the fold change equal or greater than 1). Unique HUGO identifier of differentially expressed genes were passed to the Enrichr web application (Kuleshov et al., 2016) for functional enrichment analysis. The data discussed in this publication (including raw and processed files, expression values for each gene in each sample, and fold change and adjusted *P*-value for each gene in the three comparisons: mucoid vs blood, serous vs blood, mucoid vs serous) have been deposited in NCBI's Gene Expression Omnibus [46] and are accessible through GEO Series accession number GSE125532 (https://www.ncbi.nlm.nih.gov/geo/query/acc.cgi?acc=GSE125532).

R code used to perform the analysis and produce the plot is available through a GitHub repository: https://github.com/gdevailly/oitis_array_analysis.

### RT-qPCR

Thirty-two RNA extracts were made from wbcs in COME effusion (one ear sample per child) and matching blood samples were analyzed RNA from both blood and middle ear effusion were run on custom designed 96-well TaqMan Fast array plates (Applied Biosystems) following manufacturer's instructions. There were 32 patient samples (2/32 serous, 2/32 intermediate, 2 non-classified and 26/32 mucoid) with RNA RIN scores ranging from 2.3 to 7.7; corresponding blood samples from these patients had RIN scores ranging from 7.9 to 9.0. RT-qPCR data was analyzed by ΔΔCt method. The efficiency of the assays was ensured by the use of inventory TaqMan assays.

### Selection of Appropriate Control Genes

Our set of endogenous control genes was selected from: *ACTB, B2M, HPRT1, HRAS, NRAS, PPIA*, and *TBP.* Two criteria were applied: (A) there should be no evidence of differential expression between blood and exudate at any of the control genes used; and (B) the variance of the normalization factor (calculated from the set of selected control genes) should be small. To address criterion (A), a paired t-test (paired within participants) was applied at each gene to test for non-zero mean difference in Ct value between blood and exudate; all genes apart from *HPRT1, HRAS,* and *NRAS* were significantly differentially expressed between blood and exudate at the 5% level, and so all genes except these three were excluded from further consideration. Then, to fulfill criterion (B), the variance of the normalization factor was estimated for each subset of these three genes using the methodology described by Chervoneva et al. (2010). Figures analogous to theirs led us to select an optimal subset comprising all three genes—*HPRT1, HRAS*, and *NRAS*. This subset provided a normalization factor with relatively low variance in each of our sub-experiments (plates and individual assays).

### Statistical Analysis of RT-qPCR Data

Fold changes for each patient were calculated using DataAssist software v3.0 by comparing gene expression levels of middle ear fluid using a patient's own blood as a control. Statistical significance was established using a two tailed paired student t-test with *P*-values adjusted using the Benjamin-Hochberg false discovery rate.

### VEGF Protein Assay

Thirty-seven COME effusion samples (one ear sample per child) and matching plasma samples were analyzed for VEGF. Tubes containing samples were weighed and blanked against an empty tube to estimate fluid volume. Depending on sample weight and viscosity the samples were diluted with 0.5, 1.0, 1.5, or 2.0 ml of PBS dispersed by vortexing for 1 min then pelleted at 3000 rpm for 10 min at 4°C. Supernatant COME effusion samples and corresponding patient serum samples were assayed for VEGF protein using MSD® MULTI-ARRAY® Human VEGF assay (Meso Scale Discovery). The COME effusions data was normalized to plasma volume by multiplying the COME assay value by its dilution (weight of glue and volume of PBS diluent). The assay read out is calibrated against standard curve and VEGF titres are in units of pg/ml.

VEGF titres in effusion and plasma titres were not normally distributed (*P* ≥ 0.01; D'Agostino & Pearson omnibus normality tests) and were therefore analyzed with a Wilcoxon matched-pairs signed rank test. We performed a Spearman correlation analysis between patient age and COME VEGF titre. Data were graphed using Prism Graph Pad.

## Results

### Patients and Patient Samples

The patient cohort consisted of children undergoing grommet surgery for COME. There were 12 girls age range 1.4–8.0 years of age (4.93 ± 0.57 mean ± SEM) and 40 boys age range 3.1–9.4 (5.36 ± 0.25). The average age of the girls and boys was not significantly different (*P* = 0.50; 2 tailed t-test with unequal variance).

Effusion samples were characterized as serous, mucoid or intermediate in consistency and whether or not blood-staining was present (see *Methods and Materials* for details). The frequency of unilateral glue ear was not significantly different in boys (8/40) and girls (2/12) (*P* = 1.0, Fisher exact). The frequencies of each sample category were not significantly different in boys (17/69 serous, 10/69 intermediate, 42/69 mucoid; 3 samples were too small to assess) and girls (3/22 serous, 1/22 intermediate, 18/22 mucoid; *P* = 0.30, Fisher Exact).

The volume and quality of COME samples varied and not all were suitable for analysis. We found it impractical to split COME effusion samples at the time of collection or subsequently in the lab, so contralateral ear samples from each child were used for either RNA or for protein analysis. Whole blood was collected into two tubes, one for RNA, a second for plasma.

The breakdown of samples used in each assay can be summarized as follows. Thirty-two matched pairs of COME effusion (one sample per child from either the right or left ear) and blood samples were analyzed by RTqPCR; 37 matched pairs of COME effusions samples (one ear sample per child) and plasma were analyzed for VEGF protein. Transcriptome analysis was performed on 6 serous COME effusions from six children and 6 mucoid effusions from five children (samples from both ears in one child), and their matching 11 blood samples. Forty-eight cytology smears were made from 30 children (one ear sample per child), and 9 children (samples from both ears).

### Glue Ear Cytology

Forty-eight samples with mucoid n = 31, intermediate n = 8, and serous n = 9 gross appearance yielded good quality slide preparations for full cytological examination. Cytological categorization of mucus was graded as heavy, intermediate or low/absent which we hereafter refer to as mucoid, intermediate and serous. See **Table S1** for cytology grading classification.

**Figure 1:**
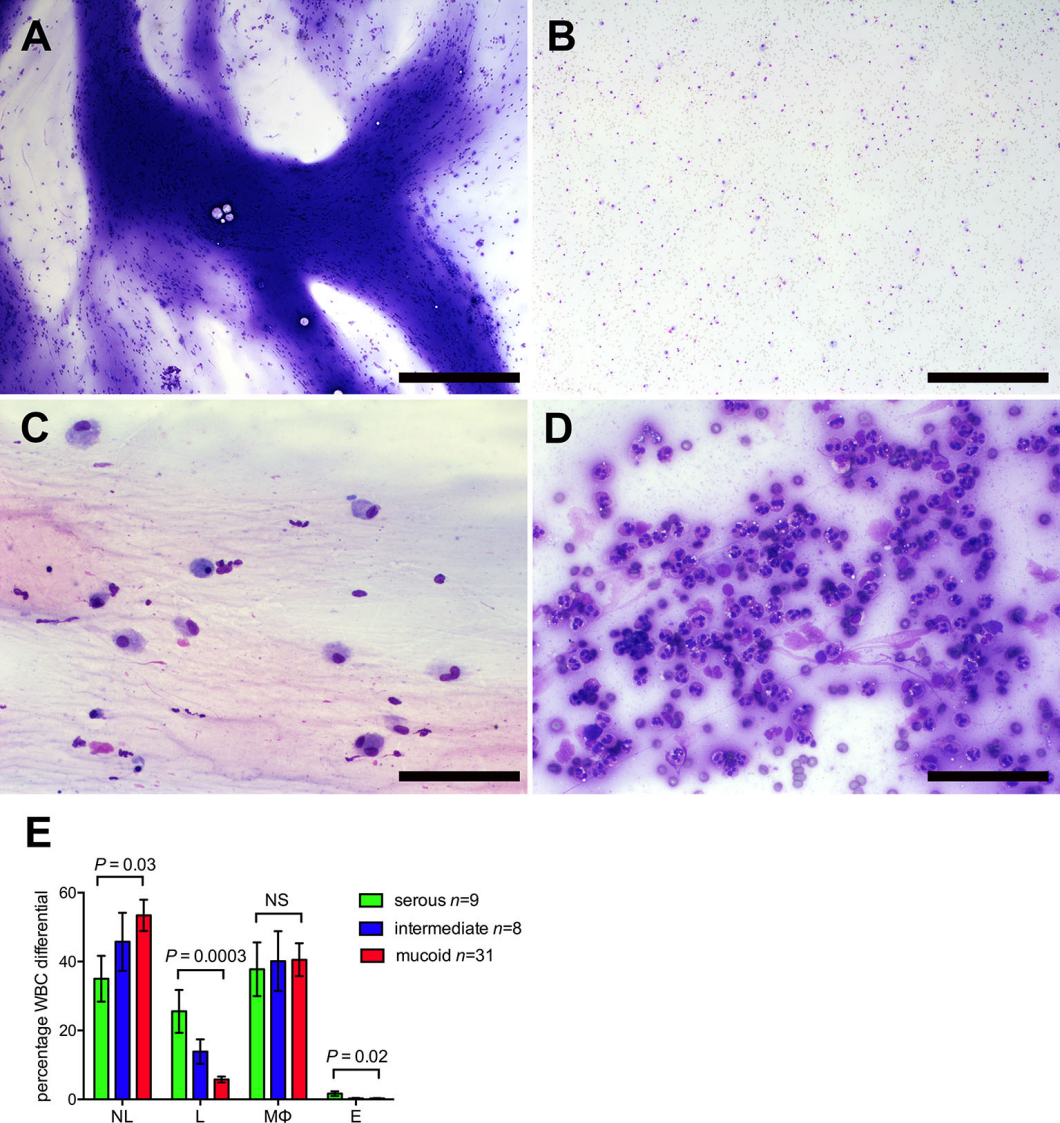
Glue ear cytology. Examples of different cytological appearance of the glue ear samples graded on an ordinal scale 1–3 (See **Table S1** for cytology grading criteria). **(A)** Sample with high mucus score and moderate cellularity. **(B)** Sample with low mucus score and moderate cellularity. **(C)** Sample with low cellularity and high proportion of macrophages (75%) **(D)** Sample with high cellularity and high proportion of neutrophils (94%). May-Grünwald Giemsa stained direct smear preparations. Scale bars **(A, B)** 500 μm, **(C, D)** = 100 μm. **(E)** Cytology differentials for serous, intermediate and mucoid samples. Mixed statistical models were used to compare serous and mucoid samples (see Methods and Materials). NL, neutrophil leukocytes; L, lymphocytes; MØ, macrophages; NS, probability not significant P > 0.05.

COME samples showed varying degrees of cellularity and cellular composition, but leukocytes largely predominated in all samples and only occasional suspected epithelial cells were noted. Cellularity appeared slightly higher in mucoid samples (**Table S2**), however a more precise quantification (e.g. cell counts on the fluid) rather than the estimated grading from the smears would be needed to better investigate this finding. Neutrophils and macrophages predominated in all sample classes. However, serous samples had significantly higher lymphocyte and eosinophil differential counts, while mucoid samples had significantly higher neutrophil differential counts. Macrophage differentials did not differ significantly between serous, intermediate and mucoid samples (**Figure 1**). Occasional macrophages showing erythrophagocytosis were observed in low numbers of samples (12.5%); this may suggest that in those cases the presence of RBCs may not only be due to iatrogenic contamination during the sampling procedure, but minimal true hemorrhage may have occurred. Occasional columnar epithelial cells were observed in 14.6% of samples.

We explored associations for cytology features with patient metadata (age, racial ethnicity, duration of disease, vaccination history, antibiotic use, previous grommet surgery, occurrence of atopy). Blood stained effusions were associated with higher levels of lymphocytes, mucus and red blood cell count, and macrophage erythrophagocytosis was higher in serous than mucoid effusions. Male children had significantly more serous effusions as well as higher lymphocytes (**Table S2**). Additional analyses were performed with adjustment for blood-stained effusions and for gender, which confirmed the differences in leucocytes between mucoid and serous effusions were not confounded by these factors.

Other borderline significant associations which may warrant further investigation are listed in **Table S2**.

### Microarray Analysis

To gain a better understanding of gene expression profiles of mucoid and serous effusions we performed microarray analysis of wbc RNA extract from effusions with a high RNA yield: Transcriptome analysis was performed on 6 serous COME effusions from six children and 6 mucoid effusions from five children (samples from both ears in one child), and their matching 11 blood samples. We used the Affymetrix Human Gene 2.0 ST microarray, using annotations provided by BrainArray (Molecular and Behavioral Neuroscience Institute, University of Michigan) (Xuan et al., 2010). After data normalisation using AromaAffymetrix (http://aroma-project.org/), we performed a multidimensional scaling of the data (**Figure S1**).

**Figure 2:**
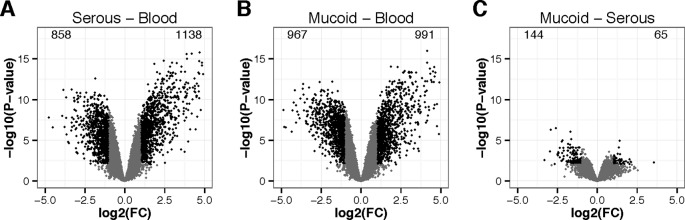
Volcano plots of differentially expressed genes in wbcs. **(A)** middle ear serous effusion versus blood, **(B)** middle ear mucoid effusion versus blood, and **(C)** mucoid versus serous effusions. X-axis represent the log2 of the fold change (FC). Y-axis represents –log10 of the *P*-value of the difference. Black points represent genes with a fold change greater than 2 or lower than 0.5 and a *P*-value lower than 0.01.

Dimension 1 clearly discriminates blood from ear effusions, while dimension 2 discriminates the different ear effusions, notably but incompletely splitting mucoid from serous effusion. No association from pairs of effusion—wbc samples was detectable neither on dimension 1 nor on dimension 2. Thus we performed a differential gene expression analysis by comparing all mucoid effusion samples against all blood samples, all serous effusion samples against all blood samples, and all mucoid effusion samples against all serous effusion samples (**Figure 2**). As expected from the multidimensional scaling, more genes were differentially expressed between mucoid effusion and blood, and between serous effusion and blood, than between mucoid and serous effusions. Due to our experiment design, differences in gene expression may be the results of difference in wbc populations as well as difference in gene expression in one or several cell types.

**Figure S2** represents a heatmap view of all differentially expressed genes identified from the microarrays, using as a threshold a minimum absolute log2 fold change (FC) of 1, and a maximal adjusted *P*-value of 0.01 (FDR correction for multiple testing). It shows that most of the differences observed in gene expression were shared in serous and mucoid effusion as compared to blood. **Table S4** presents limma gene expression data for mucoid COME effusion and blood; serous COME effusion and blood; and mucoid COME effusion and serous COME effusion. We note that there is differential expression of epithelial genes such as MUC5B that is significantly higher (adjusted value *P* = 0.0354) in mucoid versus serous COME samples, which we attribute as originating from the small numbers of columnar epithelial cells that are found in COME effusions.

Functional enrichment of the differentially expressed genes using Enrichr (Chen et al., 2013; Kuleshov et al., 2016) reveals characteristics of the effusion of COME patients (**Table 1**). It shows that the effusion is an inflammatory and hypoxic environment, enriched in CD33+ myeloid cells (granulocyte and macrophage lineages) and CD14+ monocyte cells. Its transcriptional signature overlaps those of RELA, SOX2, and MITF (amongst other) transcription factors.

**Table 1:** List of enriched categories in genes upregulated in middle ear effusions as compared to blood, as identified with the Enrichr web tools.

	Enrichr database	Dataset	Mucoid vs blood *P*-value	Serous vs blood *P*-value
Ontologies and pathways	WikiPathways 2015	Toll-like receptor signaling pathway (Homo sapiens)	0.001995	0.005231
		RANKL/RANK signaling Pathway (Homo sapiens)	0.0006495	0.005231
	BioCarta 2015	hypoxia-inducible factor in the cardiovascular system	0.001801	0.006227
		classical complement pathway	0.005481	0.004845
	GO Biological Process	inflammatory response (GO:0006954)	7.78E-08	0.000002643
		response to hypoxia (GO:0001666)	6.61E-07	0.00009897
		regulation of leukocyte activation (GO:0002694)	0.00002025	8.04E-08
Enriched cell types	Human Gene Atlas	CD33+_Myeloid	0.000008913	0.028
		CD14+_Monocytes	0.01814	0.07511
	Mouse Gene Atlas	macrophage_peri_LPS_thio_0hrs	3.42E-13	1.15E-11
Regulation	ChEA	RELA-24523406-FIBROSARCOMA-HUMAN	6.51E-88	2.94E-49
		SOX2-20726797-SW620-HUMAN	2.03E-79	3.70E-73
		MITF-21258399-MELANOMA-HUMAN	1.11E-141	5.50E-154
		ZNF217-24962896-MCF7-HUMAN	4.39E-67	7.37E-67
		NR1H3-23393188-ATHEROSCLEROTIC-FOAM-HUMAN	2.11E-50	2.13E-49
		TP63-23658742-EP156T-HUMAN	3.01E-83	2.38E-75
	TRANSFAC and JASPAR PWMs	FOXC1 (human)	5.74E-16	4.60E-17
		TCF4 (human)	1.14E-18	1.27E-18
		JUN (human)	9.37E-16	1.20E-18
		ETS1 (human)	3.32E-14	5.51E-16
		GATA2 (human)	1.81E-13	3.77E-14
	ENCODE TF ChIP-seq 2015	IKZF1_GM12878_hg19	4.28E-28	3.94E-25
		SMARCC1_HeLa-S3_hg19	3.14E-18	2.57E-20
	NCI-Nature	HIF-1-alpha transcription factor network	4.78E-07	0.000002112
		HIF-2-alpha transcription factor network	0.004811	0.07746
	Single Gene Perturbations from GEO up	RARA Activation - AM580 (RARA Agonist) human GSE5679 sample 2385	2.55E-67	4.65E-55

Differences in gene expression were observed when comparing mucoid against serous effusion RNA extracts (**Table S3**). For example, IL1RN, SERPINE1 were more expressed in mucoid fluid, while CXCL9, CXCL10, CXCL11, CXCL12, CD3D, CD96, IL32, and APOBEC3G were significantly more expressed in serous fluids (**Table S4**). Functional enrichment of the differentially expressed genes using Enrichr reveals that serous fluid had a transcriptomic signature enriched in CD8+, CD4+, and CD56+_NK cells and had a stronger T cell signature whereas mucoid samples had borderline enrichment in myeloid cells and monocytes (**Table S3**).

### RTqPCR Analysis Shows Upregulation of Hypoxia Signaling Pathways in COME Effusions

Thirty-two RNA wbc extracts were made from COME effusion (one ear sample per child) and matching blood samples were analyzed for 52 selected genes by RTqPCR. This confirmed significant upregulation of hypoxia signaling pathway genes including VEGF ligands (*VEGFA, VEGFB*) receptors (*KDR, FLT1*) adapter proteins (*NRP1, NRP2*), and HIF transcription factor (*HIF1A*) in COME relative to blood (**Figure S3**).

Overall there was reasonably good concordance between RTqPCR and microarray data as 22 over 27 (81%) of the differentially expressed genes according to the array were also differentially expressed according to RTqPCR experiment (*P*-value < 0.01, **Figure S3**). RTqPCR identified 14 additional genes differentially expressed in wbcs from COME effusion and blood that were not differentially expressed in our microarray analysis, suggesting that in our experimental design RTqPCR was more sensitive than microarrays, albeit with more variability.

### VEGF Protein Is Elevated in COME Effusions

Thirty-seven COME effusion samples (one ear sample per child) and matching plasma samples were analyzed for VEGF.VEGF protein titer is elevated in COME fluid samples (mixed categories) (median 6,427 pg/ml, 95% CI 3,541–12,923) compared with plasma (median 69 pg/ml 95% CI 61–92; *P* < 0.00010 2-tailed Wilcoxon matched pairs signed rank test, *n*=37 pairs). It confirms at the protein level the increase of VEGF expression as observed in both micro-array and RTqPCR data. There was a small and borderline statistically significant negative correlation between COME VEGF titre and increasing child age (Spearman r −0.3241, 95% CL −0.5930 to 0.009839, n = 37 XY pairs; *P *= 0.050).

## Discussion

### The Effusion in COME Reveals Upregulation of Hypoxia Signaling Pathways

We report that hypoxia signaling pathways are upregulated in wbcs from both mucoid and serous COME fluids compared to wbcs in venous blood. Significant upregulation was shown by microarray data analysis using Enrichr annotation strategies, including ontology and pathway analysis (using the WikiPathways database and the GO Biological Processes database), and HIF-1A regulatory pathway analysis (using the NCI-Nature database, **Table 1**). Findings were confirmed by RTqPCR for selected pathway genes (VEGF ligands, VEGF receptors and adapters, and HIF transcription factor).

We also found significantly elevated VEGF protein in COME fluids when compared to plasma. Other studies have reported the presence of VEGF protein in the effusion and the middle ear mucosa of patients with COME (Jung et al., 1999; Val et al., 2016; Val et al., 2018). The present study finding of hypoxia on transcriptional profiling was the result of an unbiased approach to whole transcript analysis and provides further evidence of wbc hypoxia in human COME. We found a small and borderline statistically significant negative correlation between COME effusion VEGF protein titre with increasing child age. This small effect requires further study, but may be interpreted as a trend towards resolution of inflammation and hypoxia and improved oxygenation of the bulla.

Upregulated hypoxia signaling in the bulla has been demonstrated in several animal models of otitis media: genetic mouse models of COME [*Fbxo11^Jf/+^, Mecom^Jbo/+^* (Cheeseman et al., 2011), *Tgif^−/−^* (Tateossian et al., 2013), *Nischarin ^edsn/edsn^* (Crompton et al., 2017) and *Eda^Ta^* (Azar et al., 2016)], after cauterization of the Eustachian tube in the rat (Huang et al., 2012), and that induced by intrabullar injection of Non-typeable *Haemophilus influenzae* in the mouse (Husseman et al., 2012) or gastric-content in the rabbit (Basoglu et al., 2012). This underlines the common association between hypoxia with inflammation (see below) regardless of the initiating cause of OM, duration of inflammation, effusion phenotype or species differences.

Hypoxia signaling is a common finding in inflamed microenvironments, and it is unclear whether such signaling is the cause or the result of inflammation. Surgical ventilation of the middle ear in the *Mecom^Jbo/+^* mouse model reduces tissue hypoxia and inflammation (Bhutta et al., 2014), suggesting that alleviation of tissue hypoxia may be a contributory therapeutic mechanism underlying grommet insertion for treatment of COME. In addition, administration of VEGF receptor inhibitors to young *Mecom^Jbo/+^* mice at the time of initiation of OM moderates the progression of hearing loss (Cheeseman et al., 2011). The efficacy of such treatment in chronic OM has not yet been established. It seems worthwhile to further explore the potential for targeting hypoxia pathways in chronic OM.

### Upregulation of Other Inflammatory Networks in COME

Hypoxia in mucoid and serous bulla effusions is accompanied by myeloid cell signature and upregulation of inflammatory networks (**Table 1**). Enrichr analysis revealed upregulation invoked in the Toll-like receptor (TLR) pathway, the complement pathway, and the RANK/RANKL pathway.

TLRs are components of the innate immune system, and typically respond to “pathogen associated molecular patterns” (PAMPs) on microbial cell walls or pathogen membranes. However, TLRs can also be activated by “damage-associated molecular patterns” (DAMPs): endogenous intra or extracellular proteins released as a result of cell necrosis from host tissue injury. Why TLRs respond to endogenous signals is not known, but in such circumstances TLR activation may perpetuate inflammation, risking further tissue damage. TLR activation is thought to contribute to chronic inflammation in diseases such as rheumatoid arthritis and atherosclerosis (Drexler and Foxwell, 2010), and our transcriptome data suggest TLRs are also active in chronic otitis media. A previous genetic association study found that polymorphism in the *TLR4* receptor is a risk factor for childhood COME (although this finding was not replicated) (Hafren et al., 2015), and a *Tlr* deficient mouse exhibits altered immune response to bacterial challenge of the middle ear (Tyrer et al., 2013).

Microbiome analysis of human COME samples (Liu et al., 2011; Jervis-Bardy et al., 2015; Chan et al., 2016; Krueger et al., 2017) shows the presence of diverse bacterial communities that are a potential stimulus for TLR activation along with ongoing host tissue damage. Targeting induction of TLR pathways, for example through administration of anti-microbials, specific antibodies or proteases (Piccinini and Midwood, 2010), are logical strategies to counteract this effect.

Complement consists of a cascade of innate immune proteins that respond to pattern recognition molecules displayed on the surface of potential pathogens or damaged cells, including apoptotic or necrotic host cells (Ricklin and Lambris, 2013a). In mouse models mutation at complement loci alters the duration of induced otitis media (Tyrer et al., 2013). In COME it may be that complement activation is a consequence of persistent pathogen presence, presence of necrotic or apoptotic hosts cells (as often found in a hypoxic inflammatory environment), a result of cross-talk with activated TLR pathways (Song, 2012), or via other mechanisms. Like TLR pathways, experimental data suggest that complement pathways could be antagonized with small molecules or antibodies (Ricklin and Lambris, 2013b).

The RANK–RANKL pathway affects bone remodeling and repair as well as immune regulation. This pathway mediates interactions between osteoblasts and osteoclasts as well as between T cells and dendritic cells, which in turn can affect function of fibroblasts (Walsh and Choi, 2014). The role of RANKL in inflammation is still not well understood, but is thought to be an immunoregulatory role, largely through activation of the transcription factor NFkB. Some mouse models carrying mutations at loci in the NFkB pathway develop chronic OM (Tyrer et al., 2013). Again there are compounds to target the RANK-RANKL pathway, which have previously been used to treat osteoporosis (Walsh and Choi, 2014).

Hence, our transcript analysis reveals upregulation of hypoxia, TLR, complement and RANK-RANKL pathways. It suggests that targeting one of these pathways, or perhaps targeting several of them with a cocktail of molecular inhibitors, could be a mechanism to antagonize and moderate inflammation in COME.

### Differences in Immune Cell Profiles in Mucoid and Serous COME

Cytology differentials showed neutrophils and macrophages are predominant inflammatory cell types in COME samples with heavy, intermediate, and low mucus, however mucoid samples have a significantly higher percentage of neutrophils, while serous effusions have a significantly higher percentage of lymphocytes and eosinophils. Eosinophils represent <2% of overall wbc population in our study of childhood COME. These eosinophil percentages are similar (2.6%) to older patients ~50 years-of age with COME, and contrast with much higher eosinophil differentials (13.4%) in patients with eosinophilic OM (Uchimizu et al., 2015).

While our cytological analysis did not characterize lymphocyte sub-populations, the transcript analysis indicates serous fluids have CD4+ and CD8+ T-lymphocyte, and NK cell signatures, whereas mucoid effusions have an enriched myeloid cell signature (**Table S3**). In flow cytometry analysis of COME effusions (not subdivided into mucoid and serous effusions), CD4+ and CD8+ T-lymphocytes, but not NK cells, were elevated with respect to blood populations (Skotnicka et al., 2005).

Earlier studies employing immunological techniques [e.g. cell rosetting assays (Bernstein et al., 1978)] and/or cytochemistry to characterize lymphoid cell subsets (Palva et al., 1978; Palva et al., 1980; Palva et al., 1980) emphasize the importance of lymphocytes in COME fluids. T cell differentials in serous and mucoid samples are equivalent (Palva et al., 1980), and the presence of T lymphocytes in mucoid COME is suggestive of delayed-type hypersensitivity, or mucosal injury by immune complexes (Palva et al., 1980). In another study comparing serous, seromucinous and mucoid samples recognize macrophages and lymphocytes in all samples, with serous samples containing predominantly macrophages and T cells; seromucinous samples, macrophages, T and B cells; and mucoid samples mainly B cells (Bernstein et al., 1978).

The high prevalence of neutrophils in mucoid, serous and intermediate COME effusions in this study is consistent with the proteomic findings of neutrophils and NETs described by Val et al. (2016). A subsequent proteomic analysis by these authors focusing on the differences between mucoid and serous COME samples indicated that higher neutrophil signature NET's in mucoid COME associated with greater MUC5B, and that serous COME samples have higher early innate immunity markers, complement and immunoglobulins (Val et al., 2018).

Taken together these data shows there are significant differences in the inflammatory cell profiles suggestive of an underlying difference in immunological basis for serous versus mucoid COME. There may be several contributory factors. Lim et al. (Lim et al., 1979) found that neutrophils predominated in COME effusions that had culturable pathogenic bacteria whereas lymphocytes were prominent in culture negative effusions and furthermore, variation in effusion microbiome had an impact on mucin content (Krueger et al., 2017). We did not collect sample microbiome data that would allow us to evaluate this possibility.

### Strengths and Limitations of This Study

To our knowledge this is the first reported whole genome transcript analysis of the effusion in human COME. The strengths of our study are that we had a well phenotyped patient population and, by grouping COME samples into grossly mucoid and serous we were able to perform cross-comparisons and whole genome transcript analysis revealing differences in immune cell population and gene expression. Parallel differences were observed in mucoid and serous sample cytology. Results from the microarray were validated for the hypoxia-signaling pathway with RTqPCR.

Here we analyzed transcripts from the effusion in COME without extracting or differentiating the cells within that effusion, although cytological analysis would suggest that this is largely the signature from leucocytes. Future studies may aim to analyse the transcript in middle ear mucosa. We used peripheral blood wbc as control for gene expression in bulla effusion wbc, which will constitutively be different, but absence of immune cells and fluids in the healthy non-inflamed bulla means that a better control tissue is not available. For the same reason, we used plasma as the baseline control for VEGF protein in COME fluids. There is however a rationale for comparing bulla fluids with blood as the wbcs in COME effusions will have been recruited from the blood and effusion proteins will contain a transudate of plasma. We also acknowledge the general problems with the analysis of mRNA transcriptome, in that this may not directly correlate to protein expression. The microbiota was not assayed in COME effusions and this may be a determinant of glue ear MUC5B content (Krueger et al., 2017).

## Conclusion

The cytological and transcriptome data from this study confirm that COME is an inflammatory disorder, with infiltration by a leucocyte population largely composed of neutrophils and macrophages, albeit with differences in cell populations in serous versus mucoid effusions. Transcriptome pathway analysis indicates upregulation of hypoxia signaling, the TLR pathway, complement, and the RANK-RANKL pathway. The prospects for utilizing transcriptome data to identify new targets for medical interventions depend on validation of candidate target proteins and a precise understanding of their role in effusion and mucosal inflammation. Pre-clinical animal models such as mouse models of chronic otitis media may assist future drug trials, ideally utilizing compounds that would be administered locally to the chronically inflamed middle ear.

## Data Availability Statement

The datasets generated for this study can be found in the GEO Series accession number GSE125532.

## Ethics Statement

The studies involving human participants were reviewed and approved by Oxfordshire Research Ethics Committee. Written informed consent to participate in this study was provided by the participants' legal guardian/next of kin.

## Author Contributions

MB: Clinical lead for project, initial draft of paper. JL: Consent, and initial processing of samples. LH: Study coordinator, and initial processing of samples. DW: Transcript analysis. HT: Laboratory assistance. GN: Statistical analysis. SB: Study design. HB: Statistical analysis, CP: Cytological analysis, GD: Bioinformatic analysis and initial draft of paper. JR: Lead for ethical approval of study. MC: Scientific lead for project, initial draft of paper.

## Funding

This work was funded through a Medical Research Council Technology Development Gap Fund grant A853-0092 and a BBSRC Institute Strategic Programme Grant BB/J004316/1 to the Roslin Institute. The microarray data were generated in the OXION facility at Oxford University and this was supported by Wellcome Trust grant 084655. MRC Harwell provided the open access publication costs.

## Conflict of Interest

The authors declare that the research was conducted in the absence of any commercial or financial relationships that could be construed as a potential conflict of interest.
