# Supplementary material for: Transcript Analysis Reveals a Hypoxic Inflammatory Environment in Human Chronic Otitis Media With Effusion
Source: Front Genet. 2020 Feb 21;10:1327. doi: 10.3389/fgene.2019.01327 (PMC7047850; doi:10.3389/fgene.2019.01327)
Supplement: Supplementary file 1 [file DataSheet_1.zip › Table 1 - 3.DOCX]

**Table S1** Glue ear cytology characteristics

| Characteristic | Ungraded | Grade 0 | Grade 1 | Grade 2 | Grade 3 |
| --- | --- | --- | --- | --- | --- |
| Mucus |  |  | small amount of pinkish background, no aggregates of mucus, with embedded cells | intermediate amount between 1 & 3, moderate numbers of mucus aggregates not too thick | very thick background, large number of darker aggregates of mucus and cells |
| RBC (amount of blood): RBC/40x field, average in 10 fields |  |  | <20 | 20-200 | >200 |
| Cellularity: counting both 'clumps' and spread areas, cells/40x field, average in 10 fields |  |  | <15 | 15-60 | >60 |
| Leukocyte differential count (200 cells) | % neutrophils, lymphocytes, macrophages, eosinophils, mast cells |  |  |  |  |
| Macrophages | | | | | |
| Vacuolation (VAC) |  |  | <10% | 10-50% | >50% |
| Erythrophagocytosis (EPG) |  | absent | <30% | >30% |  |
| Leukophagocytosis (LPG) |  | absent | <30% | >30% |  |
| Hemosiderin (HSID) |  | absent | <30% | >30% |  |
| Binucleation (BN) | approx % binucleated |  |  |  |  |
| Multinucleation (MN) | approx % multinucleated |  |  |  |  |
| Neutrophils | | | | | |
| Karyolysis (KLYS) |  |  | <10% | 10-50% | >50% |
| Karyorrhexis (KRX) |  |  | <10% | 10-50% | >50% |
| Pyknosis (PYK) |  |  | <10% | 10-50% | >50% |
| Phagocytosis (PHAG) |  | absent | In 1-10 cells | In >10 cells |  |

**Table S2** Associations for cytology and patient metadata

|  | **Association** | ***P*-value** | **Description** |
| --- | --- | --- | --- |
| Blood related | Lymphocytes and bloody ear | 0.03 | Lymphocytes higher in bloody ears |
|  | Mucus and bloody ear | 0.02 | Mucus score higher in bloody ears |
|  | Red blood cell count and bloody ear | 0.001 | Red blood cell count higher in bloody ears |
|  | Mucoid category and erythrophagocytosis | 0.02 | erythrophagocytosis higher in serous ears |
| Gender | Lymphocytes and gender | 0.001 | Lymphocytes higher in males |
|  | Mucoid category and gender | 0.03 | Higher proportion of serous ears in males |
| Other | Mucoid category and cellularity | 0.02 | Higher cellularity in mucoid ears |
|  | Breast feeding and mucus | 0.05 | Higher mucus score if breast fed |
|  | Side of ear and macrophage vacuolation | 0.07 | Higher macrophage vacuolation in right ear |
|  | First grommets and cellularity | 0.06 | Higher cellularity in first grommets |
|  | Age and bloody ear | 0.08 | Children with bloody ears older |
|  | Ethnicity and bloody ear | 0.06 | More bloody ears in British children |

**Table S3**: List of enriched categories in genes differentially expressed in mucoid versus serous middle ear fluids**.**

| **Up in Serous** | | | |
| --- | --- | --- | --- |
|  | **Enrichr Database** | **Dataset** | **Serous vs Mucoid p-value** |
| **Ontologies and pathways** | KEGG 2015 | cytokine receptor interaction | 0.0001207 |
|  | KEGG 2015 | natural killer cell mediated cytotoxicity | 0.002076 |
|  | KEGG 2015 | toll like receptor signalling pathway | 0.01237 |
|  | WikiPathways 2015 | TCR signalling pathway(Homo sapiens) | 0.00307 |
|  | WikiPathways 2015 | Toll-like receptor signalling pathway (Homo sapiens) | 0.005787 |
|  | BioCarta 2015 | T cell receptor signalling pathway | 0.03473 |
|  | NCI-Nature | Downstream signalling in naïve CD8+ T cells | 0.0002608 |
|  | NCI-Nature | IL12-mediated signalling events | 0.001697 |
|  | Panther | Inflammation mediated by chemokine and cytokine signalling pathway | 0.01599 |
|  | GO Biological Process | regulation of T cell activation (GO:0050863) | 9.78E-07 |
|  | GO Biological Process | inflammatory response (GO:0006954) | 2.50E-07 |
|  | GO Molecular Function | chemokine activity (GO:0008009) | 3.26E-06 |
|  | GO Molecular Function | chemokine receptor binding (GO:0042379) | 0.000009054 |
| **Enriched cell types** | Human Gene Atlas | CD8+_Tcells | 0.00006656 |
|  | Human Gene Atlas | CD4+_Tcells | 0.008537 |
|  | Human Gene Atlas | CD56+_NKCells | 0.01355 |
|  | Mouse Gene Atlas | NK_cells | 0.01507 |
|  | Mouse Gene Atlas | mast_cells | 0.009906 |
|  | Mouse Gene Atlas | thymocyte_DP_CD4+CD8+ | 0.03132 |
| **Regulation** | ChEA | POU5F1-16153702-HESC-human | 0.000002431 |
|  | ChEA | SOX2-18358816-MESC-mouse | 0.000003636 |
|  | | | |
| **Up in Mucoid** | | | |
|  | **Enrichr Database** | **Dataset** | **Mucoid vs Serous** |
| **Ontologies and pathways** | NCI-Nature | Direct p53 effectors | 0.003617 |
| **Enriched cell types** | Human Gene Atlas | WholeBlood | 0.000159 |
|  | Human Gene Atlas | CD33+_Myeloid | 0.04799 |
|  | Human Gene Atlas | CD14+_Monocytes | 0.06239 |
| **Regulation** | ChEA | RELA-24523406-FIBROSARCOMA-HUMAN | 2.49E-11 |

**Table S4**: File lima_results.xlsx. Differential expression analysis. The three tabs corresponds to mucoid vs blood, serous vs blood, and mucoid vs serous comparison in that order. logFC: Fold change in log2 scale. AveExpr: Average expression of the gene. adj.p.Val: P value after FDR correction for multiple testing. mucoid_vs_blood is the comparison between mucoid samples over blood samples, serous_vs_blood is the comparison between serous samples over blood samples, and mucoid_vs_serous is the comparison between mucoid samples over serous samples.

The table is supplied in a separate supplementary file in excel format to enable easy data manipulation and viewing
