# Supplementary material for: Transcript Analysis Reveals a Hypoxic Inflammatory Environment in Human Chronic Otitis Media With Effusion
Source: Front Genet. 2020 Feb 21;10:1327. doi: 10.3389/fgene.2019.01327 (PMC7047850; doi:10.3389/fgene.2019.01327)
Supplement: Supplementary file 2 [file Presentation_1.pdf]

Supplementary Figures

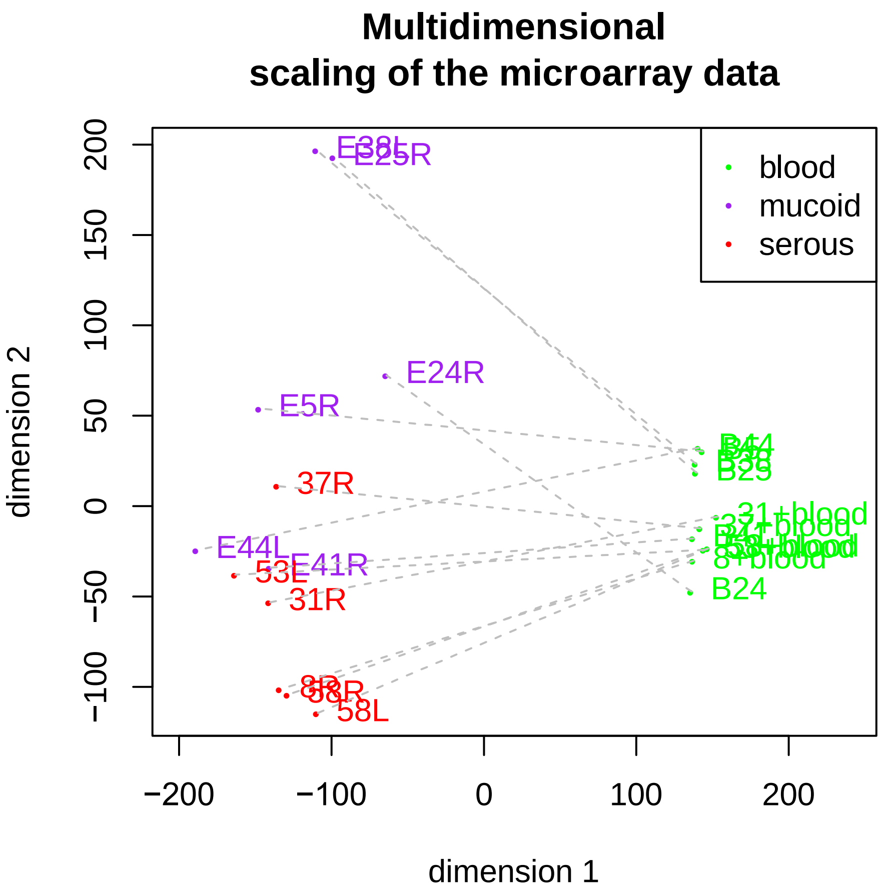

Figure S1: Multidimensional scaling of the microarray data. X-axis represents dimension 1 (74,2% of variance), Y axis dimension 2 (9.6% of variance). Green: whole blood cell RNA extract. Purple: Middle ear mucoid effusion RNA extract. Red: Middle ear serous effusion RNA extract. Grey dash lines linked pairs of samples from the same patients.

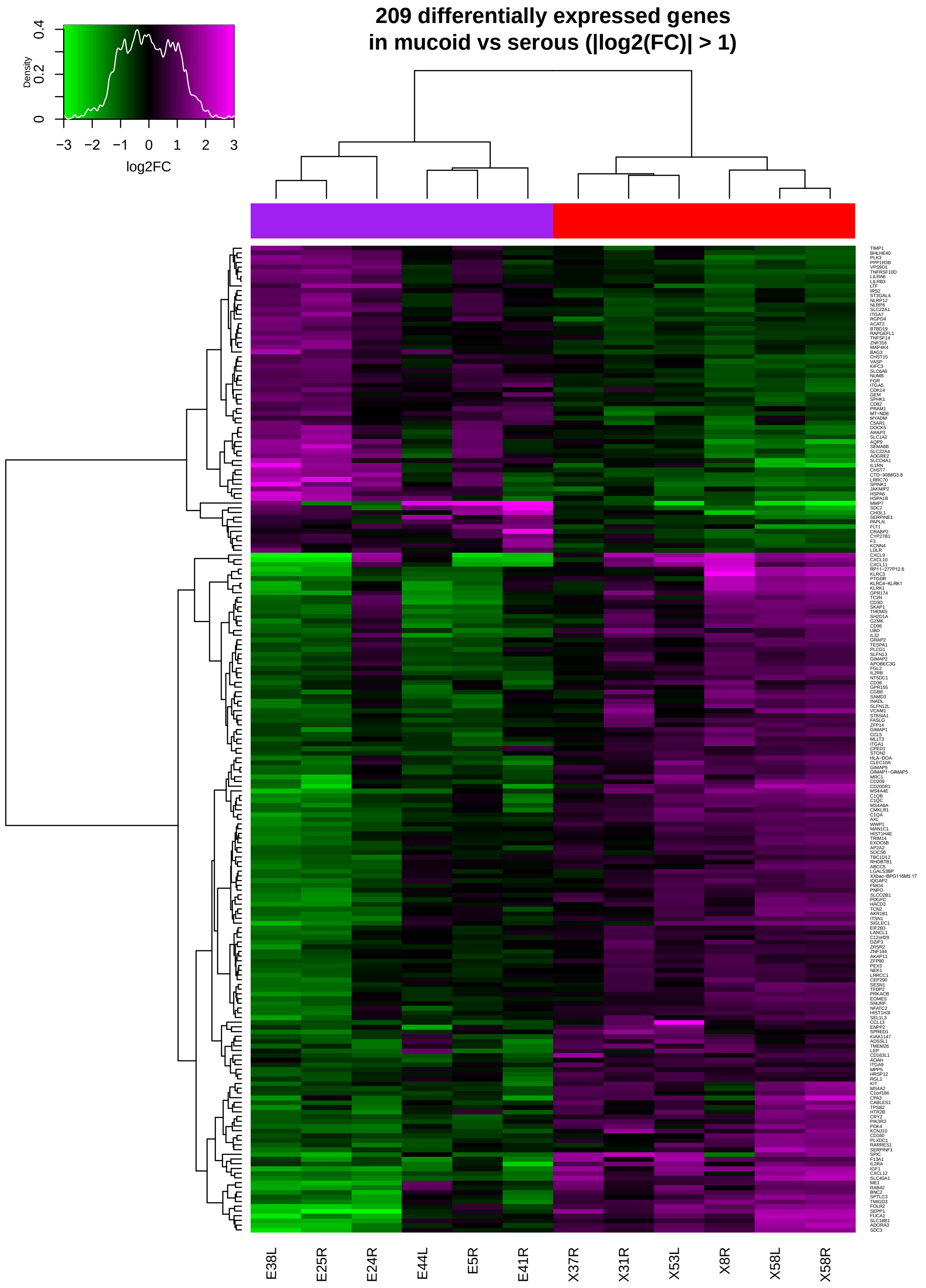

Figure S2: Heatmap of all 2682 genes differentially expressed in all three comparisons (mucoid vs blood, serous vs blood, mucoid vs serous). Green: gene is downregulated as compare to the mean of all blood extracts. Black: gene expressed similarly as the mean of all blood extracts. Magenta: gene is more expressed than the mean of all blood extracts. Purple horizontal sidebar: mucoid effusion samples. Red horizontal sidebar: serous effusion samples. Green horizontal sidebar: blood extracts. Red vertical sidebar: gene is statistically differentially expressed in mucoid versus serous effusion.

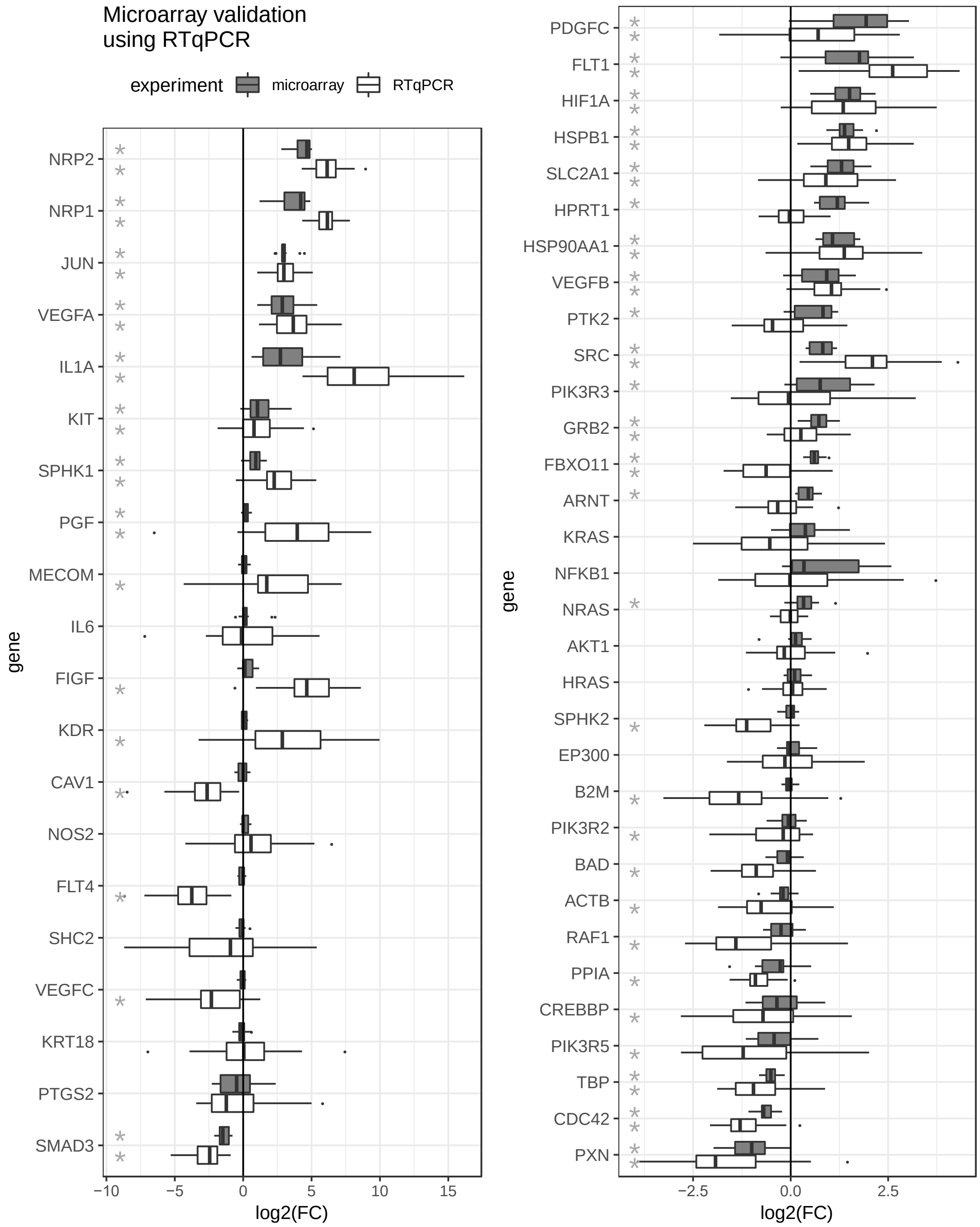

Figure S3: Comparison of microarray data and RTqPCR data for 52 genes. Gray boxplot: microarray data. White boxplot: RTqPCR data on a different set of samples. FC: Fold change of all ear effusion samples against all blood samples. *: P-value < 0.01 (student t test). For legibility reasons, data were split up in two plots of different scales.
